# A Prognostic Signature for Clear Cell Renal Cell Carcinoma Based on Ferroptosis-Related lncRNAs and Immune Checkpoints

**DOI:** 10.3389/fgene.2022.912190

**Published:** 2022-05-24

**Authors:** Yunze Dong, Ding Liu, Hongmin Zhou, Yuchen Gao, Yimingniyizi Nueraihemaiti, Yunfei Xu

**Affiliations:** ^1^ Department of Urology, Shanghai Tenth People’s Hospital, School of Medicine in Tongji University, Shanghai, China; ^2^ Department of Urology, Shanghai Tenth People’s Hospital, Nanjing Medical University, Shanghai, China

**Keywords:** ccRCC, ferroptosis, lncRNA, immune checkpoints, prognosis signatures

## Abstract

**Background:** Ferroptosis is a potential target for cancer therapy, and lncRNAs can also affect ferroptosis by regulating related genes. The pathogenesis of clear cell renal cell carcinoma (ccRCC) regarding the regulation of ferroptosis by lncRNAs is still unknown.

**Methods:** We constructed a risk model based on data in ccRCC patients obtained from the TCGA database and validated the diagnostic and prognostic value of the model. In addition, immune function and immune checkpoint variability analysis validated the association of ferroptosis with ccRCC tumor immunity.

**Results:** The characteristics of ferroptosis-related lncRNAs (FRLs) were significantly correlated with the prognosis of ccRCC patients. The prognostic characteristics of FRLs were independent prognostic factors in ccRCC patients. Gene function in the high-risk group was associated with oxygen metabolic processes and immune pathways. Immune checkpoint variability analysis showed that HAVCR2, NRP1, and HHLA2 were upregulated in the low-risk group, while CD44, TNFRSF18, TNFSF14, TNFRSF8, CD276, and TNFRSF25 were upregulated in the high-risk group.

**Conclusions:** The prognostic characteristics of FRLs can effectively predict the prognosis of ccRCC patients and provide a new direction for clinical treatment.

## Introduction

Clear cell renal cell carcinoma (ccRCC) is a common tumor of the urinary tract with a high mortality rate (Moch et al., 2016; Sung et al., 2021). Currently, immunotherapy is the latest treatment for ccRCC, and immune checkpoint inhibitors (ICIs) such as anti-PD-1/PDL1 inhibitors have been gradually used in clinical practice (Gonzalez et al., 2018; Postow et al., 2018; Chen et al., 2019; Khan et al., 2019; Zhang et al., 2019; Buonerba et al., 2020). Unfortunately, some patients are not sensitive to ICIs (Carosella et al., 2015; Tan et al., 2020). Therefore, it is critical to find biomarkers and potential target drugs for the progression and prognosis of ccRCC.

Ferroptosis is a novel form of cell death caused by iron-dependent oxidative damage (Stockwell et al., 2017). The pathological mechanism is the failure of glutathione peroxidase (GPX4), which leads to the accumulation of reactive oxygen species (ROS) on membrane (Dixon et al., 2012). Studies have shown that ferroptosis involved in tumors, degenerative diseases, and ischemia-reperfusion injury (Guiney et al., 2017; Hassannia et al., 2019; Li et al., 2019; Yan and Zhang, 2019). Interestingly, lncRNAs play a pivotal role in ferroptosis. Silencing lncRNA MEG8 can induce ferroptosis of hemangioma endothelial cells and inhibit cell proliferation (Mao et al., 2018). LncRNAOIP5-AS1 inhibited ferroptosis and promoted cell proliferation in prostate cancer cells exposed to cadmium (Lv et al., 2022). Ketamine can induce ferroptosis of hepatoma cells by targeting lncRNAPVT1/miR-214-3p/GPX4 (Wang et al., 2019; Bohosova et al., 2021). In glioma cells, upregulation of lncrNATMEM161B-AS1 promotes ferroptosis by sponging miR-27a-3p (Kapoor et al., 2021). In addition, lncRNARP11-89 inhibited ferroptosis and promoted the development of bladder cancer by sponging miR-129-5p (Yang et al., 2020).

The specific regulatory role of lncRNAs in ferroptosis remains to be further investigated. Moreover, how to regulate the aberrant expression of lncRNAs with ferroptosis is an urgent question to be investigated. Thus, we attempted to discover the regulatory role of lncRNAs in ferroptosis and provide the theoretical basis for its application in ccRCC.

## Methods and Materials

### Data Download and Study Design

The gene expression and clinical data were obtained from The Cancer Genome Atlas (https://www.cancer.gov/tcga), the Gene Expression Omnibus (GEO, http://www.ncbi.nlm.nih.gov/geo), and FerrDb (http://www.zhounan.org/ferrdb/) databases. Immune-related clinical data were obtained from Timer (http://timer.comp-genomics.org). OS and Disease-Free Survival (DFS) data were obtained from the GEPIA database (http://gepia.cancer-pku.cn/).

### Identification of FRGs and FRLs

A correlation coefficient filter criterion of 0.6 and a *p*-value filter criterion of 0.001 were used to screen for FRLs. Based on the expression data of FRLs with the corresponding survival data, prognosis-related FRLs were screened, and these FRLs would be involved in the prognostic model. The “limma” (Ritchie et al., 2015) packages were used to screen co-expression analysis of FRGs and lncRNA with *p* < 0.05 and log2FC > 2. The “limma” package was performed to identify FRGs (*p* < 0.05 and log2FC > 1) and identify expression level of FRG (Score Filter = 0.4, *p*-Value Filter = 0.001). The “limma” package was performed to identify FRLs (*p* < 0.05 and log2FC > 1).

### Co-Expression Network Analysis and Nomogram

COX models were constructed for lncRNAs related to survival time, survival status, and prognosis of patients. Based on the model equations, the risk scores of patients were calculated and patients were divided into high-risk and low-risk groups according to the median risk scores. The FRGs and FRLs were inputted into the STRING online tool (https://string-db.org) to construct the PPI network. Then, Cytoscape software (Shannon et al., 2003) was used to analyze the PPI network. And Cytoscape software drew the figure of FRGs and FRLs co-expression network. The “regplot” and “survival” packages were utilized to perform the nomogram.

### Cox Regression Analysis and Construction of a Proportional Hazards Model

The “limma” package was performed to identify the survival time. The “survival” and “glmnet” and “survminer” packages (Friedman et al., 2010) were utilized to perform univariate and multivariate Cox regression analyses. Through integrated Cox analysis, key FRLs were screened to construct the risk model. The risk curve was finished by the “pheatmap” treatment. The “survival”, “glmnet” and “survminer” packages were utilized to perform independent prognostic analysis.

### Survival Analysis and Decision Curve Analysis

Based on the median gene expression/risk score, ccRCC patients are classified into high and low-risk groups. Then, survival curves of OS and DFS were drawn by the “survival” and “survminer” packages in R (v.4.1.1) and GraphPad software (version 8.0). A *p* < 0.05 was considered statistically significant. Moreover, the “survival”, “survminer”, and “timeROC” packages were used to generate a time-dependent ROC curve to evaluate the predictive value of the risk model. The “survival” and “ggDCA” packages drew the decision curve.

### Gene Set Enrichment Analysis

All patients were divided into high and low-risk groups according to the median gene expression/risk score. GSEA (Subramanian et al., 2005) was performed to discover potential mechanisms and downstream signaling pathways.

### Kyoto Encyclopedia of Genes and Genomes Pathway Enrichment Analysis and Gene Ontology Analysis

The “colorspace”, “stringi”, “clusterProfiler” (Wu T. et al., 2021), “DOSE”, “org.Hs.eg.db”, and “enrichplot” packages were used to conduct KEGG enrichment analysis of FRGs and FRLs. The “colorspace”, “stringi”, “clusterProfiler”, “DOSE”, “org.Hs.eg.db”, and “enrichplot” packages were used to conduct gene ontology analysis of FRGs and FRLs. The results were visualized by the “ggplot2” package in the R program. A *p* < 0.05 was selected as the cut-off point.

### Immunohistochemistry

IHC staining data are obtained from the Human Protein Atlas website (HPA, https://www.proteinatlas.org), a database based on proteomic, transcriptomic, and systems biology data that can map tissues, cells, and organs. IHC staining of NRP1, HAVCR2, HHLA2CD44, TNFRSF18, TNFSF14, TNFRSF8, and CD276 in tumor tissues and normal tissues were obtained from HPA.

### Statistics

All data analyses were performed using the R platform or GraphPad Prism 8.0. The FRLs of different groups were measured using Kaplan-Meier log-rank test method. The FRLs between high and low-risk groups were determined using the “limma” R package. *p* < 0.05 was considered statistically significant.

## Results

### Differential Expression Analysis and Functional Enrichment Analysis

We downloaded the transcriptomic data, along with the corresponding clinical data from the TCGA database. Meanwhile, we found all the genes related to ferroptosis from the FerrDb database. By taking intersections, we screened 230 FRGs differentially expressed in ccRCC. We screened out 230 differentially expressed genes (137 upregulated, 93 downregulated). Next, we screened out 76 ferroptosis-related genes (FRGs) (42 upregulated and 34 downregulated) and 1502 ccRCC-related lncRNAs (1265 upregulated and 237 downregulated), respectively.

**FIGURE 1:**
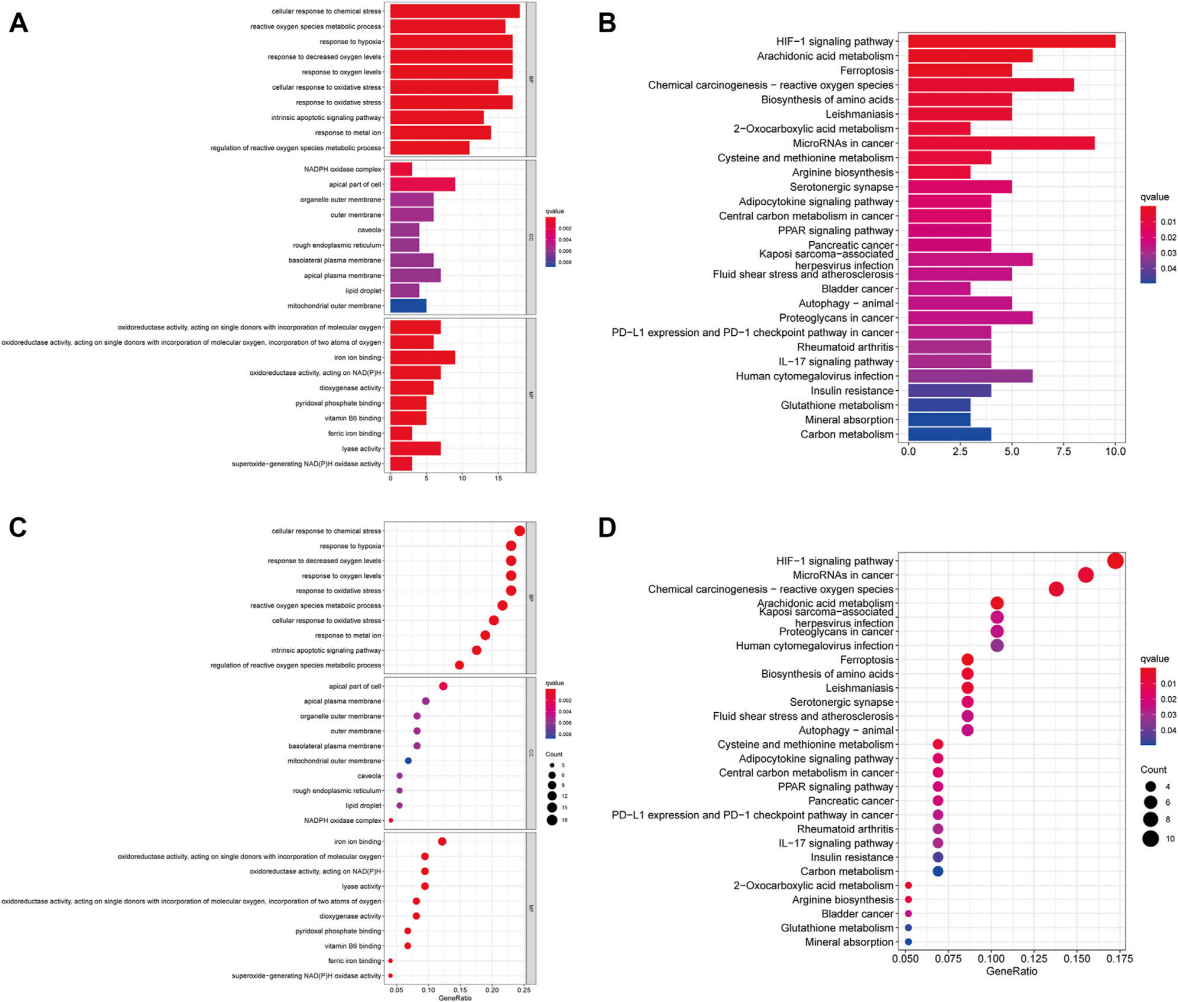
Functional Enrichment Analysis of FRGs in ccRCC patients. **(A)** The bar plot of GO functional enrichment analysis of FRGs in ccRCC patients. **(B)** The bubble diagram of GO functional enrichment analysis of FRGs in ccRCC patients. **(C)** The bar plot of KEGG pathway enrichment analysis of FRGs in ccRCC patients. **(D)** The bubble diagram of KEGG pathway enrichment analysis of FRGs in ccRCC patients (*p* < 0.05).

Then, we performed GO and KEGG pathway enrichment analysis on FRGs. In biological processes (BP), FRGs are involved in chemical stress cell response, reactive oxygen species metabolism response, low oxygen response, oxygen level response, and oxidative stress cell response. Interestingly, in molecular function (MF), they are involved in ion binding, pyridoxal phosphate binding, and vitamin B6 binding processes (Figures 1A,B). The KEGG pathway analysis also revealed that FRGs were associated with multiple signaling pathways (such as HIF1 signaling, adipocytokine signaling, and PPAR signaling) and metabolic pathways (arachidonic acid metabolism, 2-oxocarboxylic acid metabolism, cysteine, and methionine metabolism) (Figures 1C,D).

### Prognostic Signature

**FIGURE 2:**
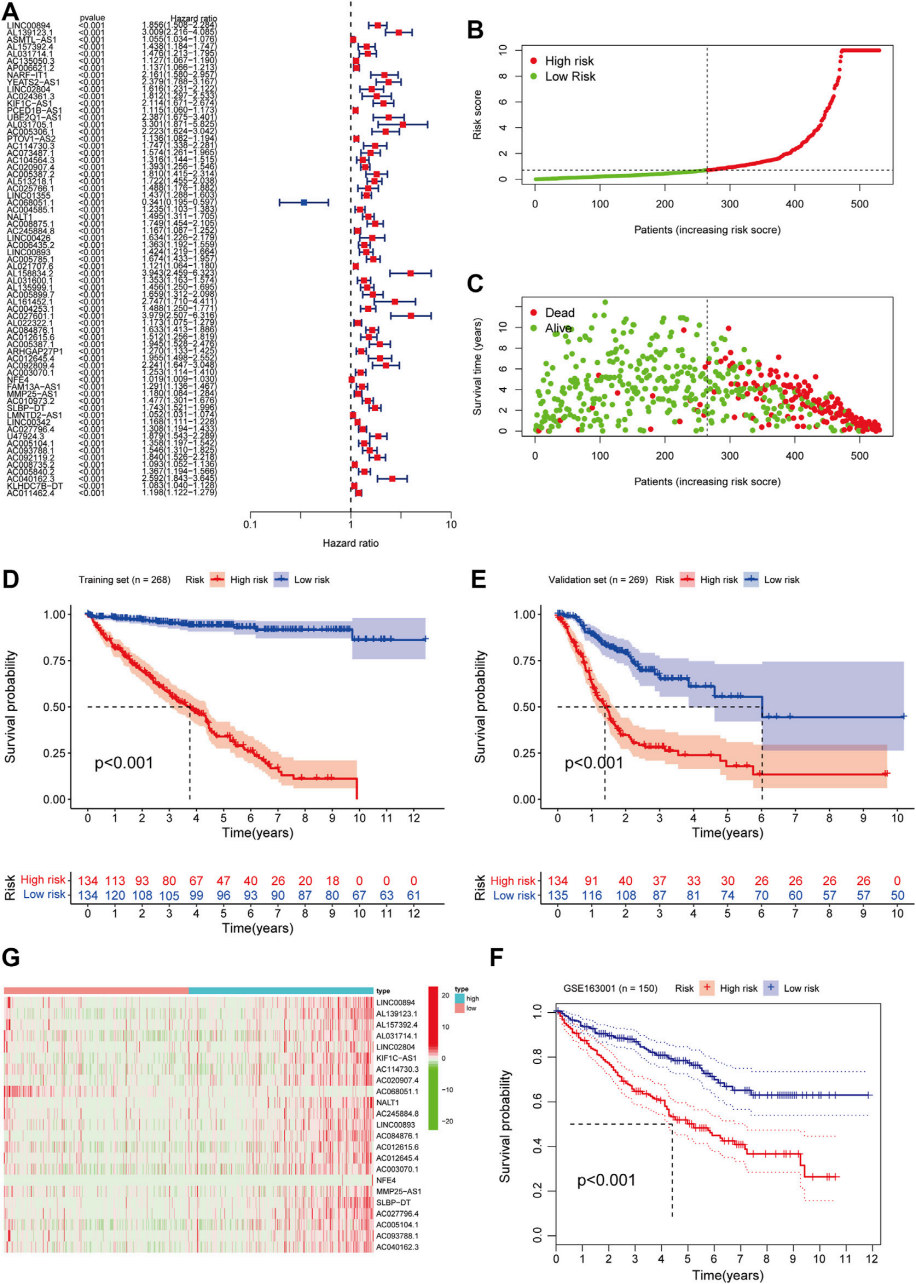
Construction and validation of FRLs Prognostic Signature in ccRCC patients. **(A)** Univariate Cox regression analysis showed that FRLs were related to the OS of ccRCC patients. **(B)** The distribution of risk scores for the high-risk and low-risk groups in ccRCC patients. **(C)** The Scatter plots of the OS in lower-risk ccRCC patients and high-risk groups. **(D)** Survival curve of the high- and low-risk groups in the training set (*n* = 268). **(E)** Kaplan-Meier survival curve of validation set (*n* = 269) comparing the high- and low-risk groups. **(F)** Kaplan-Meier survival curve analysis in the independent test set GSE163001 (*n* = 150) comparing two risk groups. **(G)** Heatmap of clinical correlation of FRLs in ccRCC. All the K-M survival analyses use log-rank tests to determine significant differences between two groups, *p* < 0.05.

From the pre-acquired data, we screened ferroptosis-related lncRNAs (FRLs) with a correlation coefficient filter criterion of 0.6 and a *p*-value filter criterion of 0.001. We then analyzed the expression data of the screened FRLs with the corresponding survival data to select prognostically relevant FRLs, which will be involved in the prognostic model. We found that 67 FRLs were involved in the OS of ccRCC (Figure 2A). We took the obtained data of FRLs and performed COX model construction on patients’ survival time, survival status, and prognosis-related lncRNAs. We calculated the risk scores of the patients and divided them into high and low-risk groups with median value: risk score = (1.546 × LINC00894 expression level) + (2.5111 × AL139123.1 expression level) + (0.0119 × ASMTL−AS1 expression level) + (0.026 × AL157392.4 expression level) + (0.9667 × AL031714.1 expression level) + (−0.32554 × AC135050.3 expression level) + 0.644 × AP006621.2 expression level) + (3.271 × NARF−IT1 expression level) + (0.142 × YEATS2−AS1 expression level) + (0.375 × LINC02804 expression level) + (0.536 × AC024361.3 expression level) + (0.15983 × KIF1C−AS1 expression level) + (−0.5863 × PCED1B−AS1 expression level) + (1.2393 × UBE2Q1−AS1 expression level) + (−2.1882 × AL031705.1 expression level) + (1.4877 × AC005306.1 expression level) + (0.12394 × PTOV1−AS2 expression level) + (-0.0795 × AC114730.3 expression level) + (1.68734 × AC073487.1 expression level) + (−0.693 × AC104564.3 expression level) + (1.1236 × AC020907.4 expression level) + (1.81 × AC005387.2 expression level) + (−0.798 × AL513218.1 expression level) + (1.4485 × AC025766.1 expression level).

We randomly assign all samples in a 1:1 ratio and divided them into training and testing datasets to construct risk features. The clinical characteristics of all patients showed in Table 1. Subsequently, we developed risk curves for both groups based on the prognostic characteristics of the FRLs, and the risk curves showed the survival status and risk scores for each ccRCC sample (Figure 2B). The scatter plot and Kaplan-Meier survival curves showed significantly higher survival rates in the low-risk group than in the high-risk group (Figures 2C,D). These results show the excellent predictive performance of the risk score model in the training set. Similarly, the prognostic value of the risk signature is verified in both the validation set and the external independent test set (Figures 2E,F). Based on the risk score, we ranked the samples and extracted the expression data of FRLs with relevant clinical information to draw a clinical relevance heat map to show the expression levels of FRLs in high- and low-risk patients. The heat map showed that 23 of the 67 FRLs were the best candidates (Figure 2G).

**TABLE 1:** The association between risk score and patients’ clinical features in the training set.

Variables	Training Set		Validation Set		*p*-Value
	(*n* = 268)		(*n* = 269)		
	No.	%	No.	%	
Age					0.812
≤65	174	64.9	178	66.2	
>65	94	35.1	91	33.8	
Stage					0.875
I	134	50.0	135	50.2	
II	29	10.8	28	10.4	
III	68	25.4	57	21.2	
IV	37	13.8	49	18.2	
T stage					0.926
T1	138	51.5	137	50.9	
T2	30	11.2	39	14.5	
T3	95	35.4	87	32.3	
T4	5	1.9	6	2.3	
N stage					0.578
N0	134	50.0	146	54.3	
N1	34	12.7	55	20.4	
N2	20	7.5	45	16.7	
N3	10	3.7	23	8.6	
M stage					0.688
M0	221	82.5	205	76.2	
M1	47	17.5	64	23.8	

### Evaluation of Independent Prognostic Factors

**FIGURE 3:**
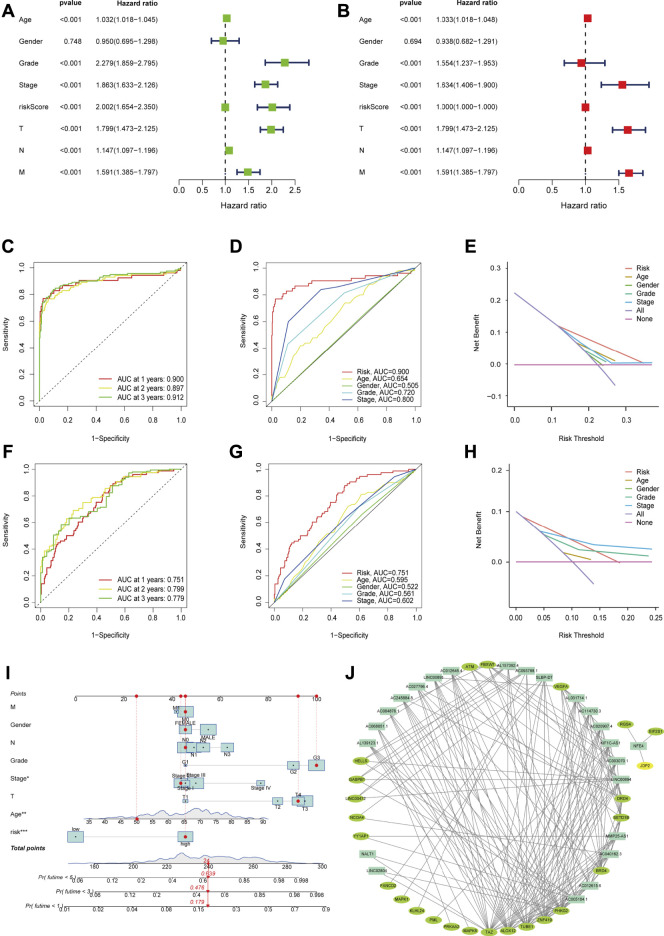
Evaluation of Independent Prognostic Factors and Construction of Nomograph **(A)** Univariate Cox regression analysis showed the correlation between overall survival rate and various clinicopathological parameters (Age, Gender, Grade, Stage, T and M stage). **(B)** Multivariate Cox regression analysis showed that age, grade, stage, and risk score were independent prognostic indicators for the overall survival of ccRCC patients. **(C)** The year 1, 2, and 3 ROC curves testified that the risk score is well predictive in the training dataset. **(D)** ROC curve analysis showed the prognostic accuracy of risk score and clinicopathological parameters in the training dataset. **(E)** The DCA showed that it was better predictive than other indicators in the training dataset. **(F)** The year 1, 2, and 3 ROC curves testified that the risk score is well predictive in the testing dataset. **(G)** ROC curve analysis showed the prognostic accuracy of risk score and clinicopathological parameters in the testing dataset. **(H)** The DCA showed that it was better predictive than other indicators in the testing dataset. **(I)** The prognostic nomogram constructed based on the risk score of FRLs and clinicopathological parameters predicted the survival rate of ccRCC patients at 1, 3, and 5 years. **(J)** A co-expression network of FRGs and FRLs.

Univariate analysis revealed that age (*p* < 0.001), stage (*p* < 0.001), and FRLs risk score (*p* < 0.001) were all independent prognostic factors for ccRCC patients, except gender (*p* = 0.748) (Figure 3A). Similarly, multivariate analysis showed a significant correlation between risk scores for FRLs and OS as well as other clinicopathological and prognostic characteristics except for gender (*p* = 0.694) (Figure 3B). In the training dataset, the ROC curves for years 1, 2, and 3 demonstrated that the FRLs risk score was strongly predictive: 1-year AUC (0.900), 2-year AUC (0.897), and 3-year AUC (0.912). Multivariate ROC curves showed that the FRLs risk score had better predictive performance than those clinicopathologic features, suggesting that the FRLs risk score is an independent predictor of survival in ccRCC patients (Figures 3C,D). The results of decision curve analysis (DCA) showed that the risk score of FRLs had better predictive power than other indicators (Figure 3E). In addition, In addition, similar results are also demonstrated in the validation set (Figures 3F–H).

### Construction of Nomograph

We integrated the prognostic features and clinicopathologic factors (age, gender, grade, and stage) of FRLs to construct nomograms to accurately estimate the survival probability in ccRCC patients (Figure 3I). Then, we constructed a co-expression network using Cytoscape software (blue nodes represent FRLs and green nodes represent FRGs) (Figure 3J).

**FIGURE 4:**
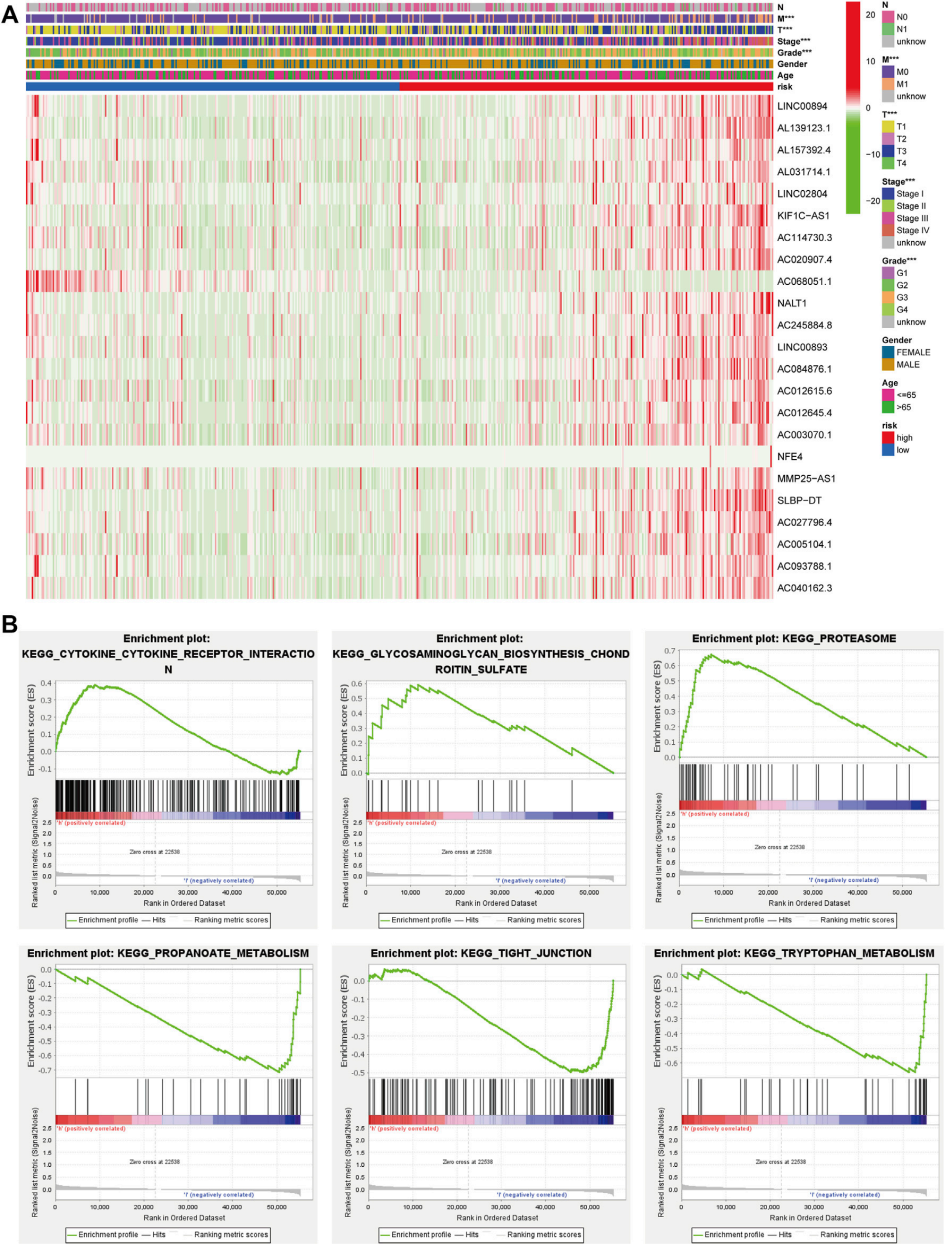
GSEA and heatmap of the clinical relevance of FRLs. **(A)** GSEA analyzed the potential downstream signaling pathways for FRLs. **(B, C)** The heatmap of the clinical relevance of FRLs.

We also plotted a heat map on the clinical relevance of FRLs (horizontal coordinates indicate samples and vertical coordinates indicate FRLs). Among the different clinical characteristics of the high and low-risk groups, several clinical characteristics of grade, T, and M stage varied significantly in both groups (Figure 4A). We identified potential downstream signaling pathways of FRLs and found that it was associated with metabolism-related pathways by GSEA (Figures 4B,C).

### Immune-Cell Correlation and Immune Function Analysis

**FIGURE 5:**
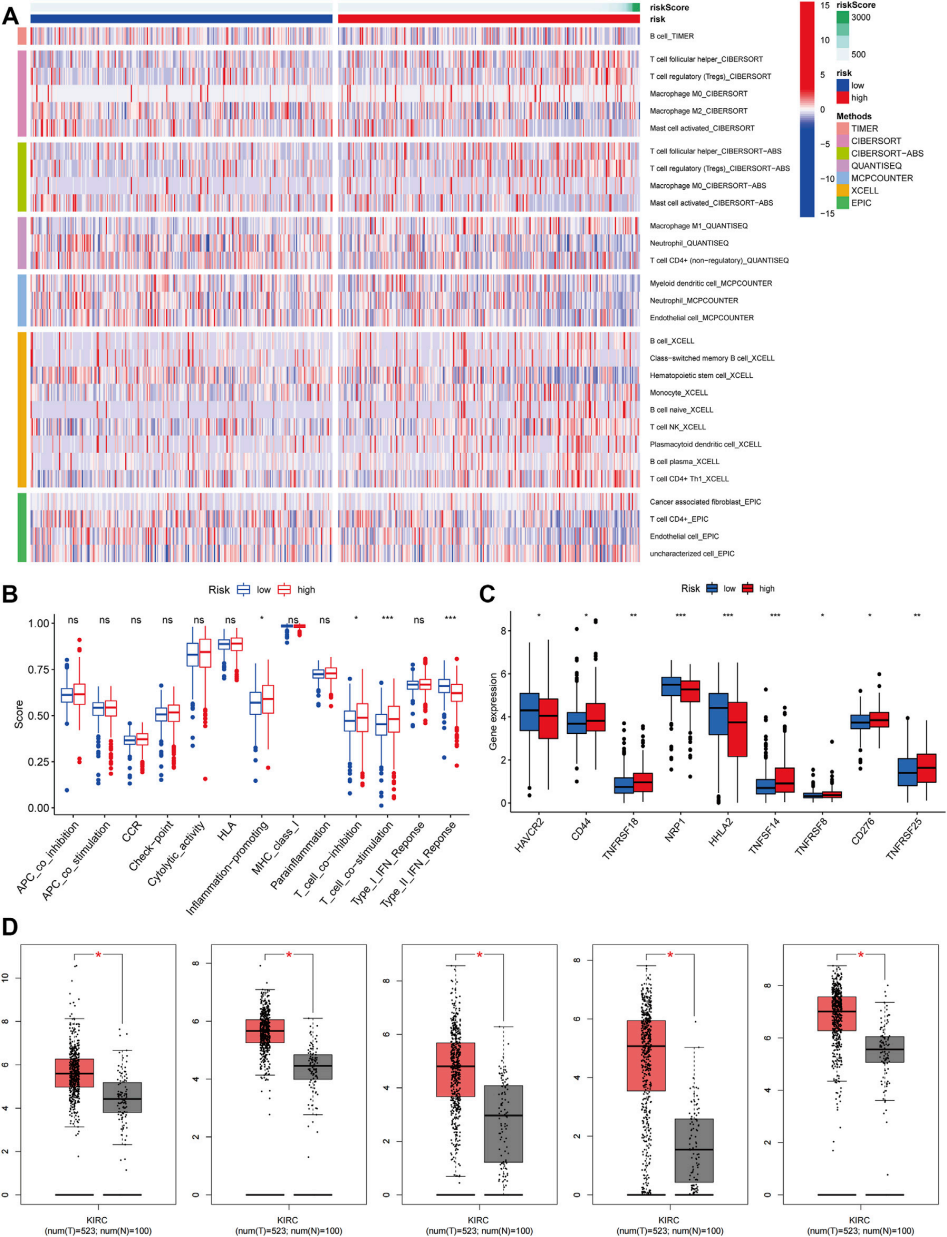
Immune-cell Correlation and Immune Function Analysis. **(A)** Heatmap showed the expression of these immune cells in high and low-risk groups. **(B)** The analysis of immune function variability of ccRCC. **(C)** The immune checkpoint differential analysis of ccRCC. **(D)** The expression level of CD44, CD276, HAVCR2, HHLA2, and NRP1 from the GEPIA database (*p* < 0.05).

We obtained immune-related clinical data from the Timer database and plotted the associated heat map (horizontal coordinates represent samples, vertical coordinates represent immune cells, and different colors represent predicted outcomes). The heat map showed that M1 macrophages, regulatory T cells, follicular helper T cells, and B cells, which are immune cells, had elevated expression in the high-risk group, while activated mast cells, neutrophils, and hematopoietic stem cells (HSC) had elevated expression in the low-risk group (Figure 5A). We then analyzed immune function variability and found that four immune functions in inflammation promotion, T cell co-inhibition, T cell co-stimulation, and type II IFN Response were actively involved in the high-risk group. These data suggest that ferroptosis is involved in ccRCC through the immune process pathway (Figure 5B).

### Immunological Checkpoint Variability Analysis

**FIGURE 6:**
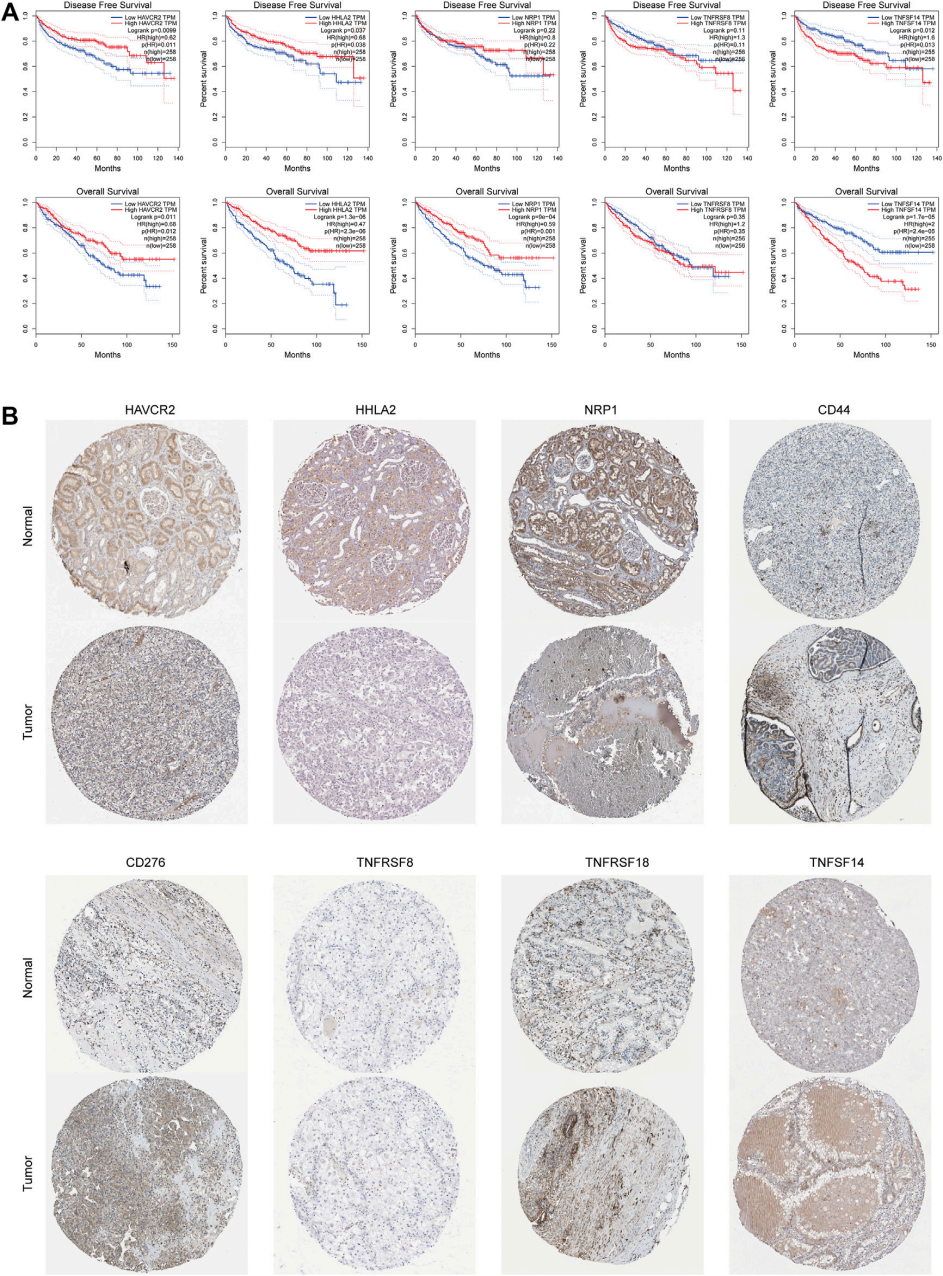
Immunological checkpoint variability. **(A)** Expression levels of TNFSF14, TNFRSF18, NRP1, HAVCR2, and HHLA2 were significantly associated with prognosis in ccRCC patients and were statistically significant on both OS and DFS. **(B)** Verify the translational expression of the immune checkpoints genes in ccRCC and normal kidney tissues.

We performed an immune checkpoint differential analysis and found that three genes, HAVCR2, NRP1, and HHLA2, were highly expressed in the low-risk group, and six genes, CD44, TNFRSF18, TNFSF14, TNFRSF8, CF276, and TNFRSF25, had increased expression in the high-risk group (Figure 5C). Then, we verified that the expression levels of CD44, CD276, HAVCR2, HHLA2, and NRP1 were statistically significant in ccRCC from the GEPIA database (Figure 5D). The expression levels of TNFSF14, TNFRSF18, NRP1, HAVCR2, and HHLA2 were significantly associated with the prognosis of ccRCC patients, and their OS and DFS were statistically significant (*p* < 0.05, Figure 6A). Meanwhile, we show the IHC staining of NRP1, HAVCR2, HHLA2CD44, TNFRSF18, TNFSF14, TNFRSF8, and CD276 in tumor tissues and normal tissues (Figure 6B).

## Discussion

Ferroptosis is involved in the occurrence, development, and progression of tumors, which aroused our interest in studying its mechanism in ccRCC (Hambright et al., 2017; Stockwell et al., 2020; Ye et al., 2020). Tumor immunology, as an emerging research field, greatly complements and improves the research system of oncology (Galon and Bruni, 2020). After tumor cells escape the surveillance of the body’s immune system under the action of various factors, they can rapidly divide and proliferate in the body, accelerating tumor deterioration, which is immune escape (Dunn et al., 2002). Immune checkpoint blockade therapy based on programmed death receptors and their ligands enhances the aggressiveness of the host immune system against tumor cells by inhibiting the binding of them (Dunn et al., 2004; La-Beck et al., 2015; Postow et al., 2018; Khan et al., 2019). Non-specificity and drug resistance are the main problems faced by conventional treatments for ccRCC (Gai et al., 2020; Zou et al., 2021). And the role of lncRNA in cancer is receiving increasing attention from researchers (Inthagard et al., 2019; Resch et al., 2021).

We highlighted the lncRNAs associated with ferroptosis and ccRCC, which may yield promising results in cancer treatment. We validated the accuracy of the FRLs risk score in predicting the prognosis of ccRCC. Next, we tested the model with ROC curves and found that it applies to different situations. The prognostic characteristics of FRLs are an independent predictor of survival in patients with ccRCC and that they can be used as multiple indicators to diagnose or predict the onset or progression of ccRCC. We integrated the prognostic characteristics of FRLs with clinicopathological factors such as age, gender, grade, and stage and constructed a nomogram to predict the probability of survival in ccRCC patients.

LncRNAs regulate abnormal tumor lipid metabolism, thereby exerting oncogenic effects in tumorigenesis, affecting tumor cell proliferation, apoptosis, migration, invasion, and ferroptosis. LncRNA can also enable ferroptosis to produce an apoptosis-independent form of cell killing. Recent studies have found that the lncRNA SNHG12/SP1/CDCA3 axis promotes progression and sunitinib resistance in RCC, which provides a new therapeutic target for sunitinib-resistant RCC (Liu et al., 2020). In addition, lncRNA 00312 inhibits RCC proliferation and invasion and promotes apoptosis in RCC by inhibiting miR-34a-5p and overexpressing ASS1 (Zeng et al., 2020). SNHG17/miR-328-3p/H2AXaxis may be involved in RCC progression, providing a potential therapeutic target for RCC (Wu J. et al., 2021). Thus, we believe that targeting lncRNAs and combining both immune checkpoints and FRLs prognostic signature may create new opportunities for the treatment of ccRCC.

However, we still have several issues to resolve. First, identifying the most significant lncRNAs associated with ccRCC remains challenging. Our study lacks the necessary experimental validation related to lncRNA expression and other relevant experimental validation. In addition, lncRNAs are generally poorly conserved across species compared to protein-coding genes. Therapeutic strategies developed based on cellular and animal models are still a long way from clinical application and may require further research.

## Conclusion

In conclusion, future research should reveal the relationship between each lncRNAs and ferroptosis and develop appropriate model systems to help the diagnosis, treatment, and prognosis of ccRCC. As the research continues, lncRNA-based therapeutic strategies will hopefully improve the prognosis of ccRCC patients.

## Data Availability

The original contributions presented in the study are included in the article/Supplementary Material, further inquiries can be directed to the corresponding author.
